# Draft Genome Sequences of *Methanoculleus horonobensis* Strain JCM 15517, *Methanoculleus thermophilus* Strain DSM 2373, and *Methanofollis ethanolicus* Strain JCM 15103, Hydrogenotrophic Methanogens Belonging to the Family *Methanomicrobiaceae*

**DOI:** 10.1128/genomeA.00199-16

**Published:** 2016-03-31

**Authors:** Takashi Narihiro, Hiroyuki Kusada, Yasuko Yoneda, Hideyuki Tamaki

**Affiliations:** a Bioproduction Research Institute, National Institute of Advanced Industrial Science and Technology (AIST), Tsukuba, Ibaraki, Japan; b Biotechnology Research Institute, The University of Tokyo, Bunkyo-ku, Tokyo, Japan

## Abstract

The family *Methanomicrobiaceae* comprises hydrogen- and formate-utilizing methanogens. Genome sequencing of nine species of *Methanomicrobiaceae* has been conducted so far. Here, we report three additional draft genome sequences of *Methanomicrobiaceae*, those of *Methanoculleus horonobensis* JCM 15517 (=T10^T^), *Methanoculleus thermophilus* DSM 2373 (=CR-1^T^), and *Methanofollis ethanolicus* JCM 15103 (=HASU^T^).

## GENOME ANNOUNCEMENT

Members of the family *Methanomicrobiaceae* in the phylum *Euryarchaeota* are known to contain hydrogen- and formate-utilizing methane-producing archaea (1). Some of the known species of this family can also use a secondary alcohol (such as ethanol, 1-propanol, and 1-butanol) as a methanogenic substrate. Currently, complete or draft genome sequences are available for nine species of *Methanomicrobiaceae* (2–6). To compare the genomic features of *Methanomicrobiaceae* species, we additionally sequenced three genomes, those of *Methanoculleus horonobensis* strain JCM 15517 ^T^ (=strain T10^T^), *Methanoculleus thermophilus* strain DSM 2373 (=CR-1^T^), and *Methanofollis ethanolicus* strain JCM 15103^T^ (=HASU^T^). These strains were isolated from a variety of methanogenic ecosystems: strain T10^T^ was isolated from a groundwater sample collected from a deep diatomaceous shale formation (7), strain CR-1^T^ was isolated from coastal sediment underlying high-temperature effluent from nucleic power plants (8), and strain HASU^T^ was isolated from a mud sample from a lotus field (9). Such ecological diversification may affect the genomic features involved in methanogenesis and interaction with syntrophic metabolizers (syntrophs).

Strains T10^T^ and HASU^T^ were provided by the RIKEN BRC through the National Bio-Resource Project of the MEXT, Japan. Strain CR-1^T^ was provided by Deutsche Sammlung von Mikroorganismen und Zellkulturen (DSMZ), Germany. Whole-genome shotgun sequencing was conducted using the Illumina MiSeq platform (Illumina, San Diego, CA, USA) at the Fasmac Co., Ltd. (Atsugi, Japan). We constructed and sequenced paired-end libraries (4,506,900, 5,396,179, and 9,926,795 bp for T10^T^, CR-1^T^, and HASU^T^, respectively) on the MiSeq. Assembly of the output reads was performed using a SPAdes software version 3.5.0 (10), and the assembled data provide >1,000× coverage of each genome. The draft genome sequences of strains T10^T^, CR-1^T^, and HASU^T^ are composed of a total of 15, 24, and 3 scaffolds, have a G+C content of 62.0, 59.3, and 60.3%, estimated genome sizes of 2.49, 2.22, and 2.75 Mb, and 2,430, 2,249, and 2,651 protein-coding genes with function prediction annotated by the Prokka pipeline (11), respectively. Further attempts to identify hydrogenotrophic methanogenesis pathways and energy conservation systems for comparison with the genomes of other *Methanomicrobiaceae* methanogens are now in progress.

### Nucleotide sequence accession numbers.

This draft genome sequence has been deposited at DDBJ/GenBank/EMBL under the accession numbers BCNY01000001 to BCNY01000015 for *M. horonobensis* strain JCM 15517, BCNX01000001 to BCNX01000023 for *M. thermophilus* strain DSM 2373, and BCNW01000001 to BCNW01000003 for *M. ethanolicus* strain JCM 15103.

## References

[1] Liu YC, Whitman WB 2008 Metabolic, phylogenetic, and ecological diversity of the methanogenic archaea. Ann N Y Acad Sci 1125:171–189. doi:10.1196/annals.1419.019. 18378594

[2] Maus I, Wibberg D, Stantscheff R, Eikmeyer FG, Seffner A, Boelter J, Szczepanowski R, Blom J, Jaenicke S, König H, Pühler A, Schlüter A 2012 Complete genome sequence of the hydrogenotrophic, methanogenic archaeon *Methanoculleus bourgensis* strain MS2^T^, isolated from a sewage sludge digester. J Bacteriol 194:5487–5488. doi:10.1128/JB.01292-12. 22965103 PMC3457187

[3] Göker M, Lu M, Fiebig A, Nolan M, Lapidus A, Tice H, Del Rio TG, Cheng JF, Han C, Tapia R, Goodwin LA, Pitluck S, Liolios K, Mavromatis K, Pagani I, Ivanova N, Mikhailova N, Pati A, Chen A, Palaniappan K, Land M, Mayilraj S, Rohde M, Detter JC, Bunk B, Spring S, Wirth R, Woyke T, Bristow J, Eisen JA, Markowitz V, Hugenholtz P, Kyrpides NC, Klenk HP 2014 Genome sequence of the mud-dwelling archaeon *Methanoplanus limicola* type strain (DSM 2279^T^), reclassification of *Methanoplanus petrolearius* as *Methanolacinia petrolearia* and emended descriptions of the genera *Methanoplanus* and *Methanolacinia*. Stand Genomic Sci 9:1076–1088. doi:10.4056/sigs.5138968. 25197484 PMC4149034

[4] Brambilla E, Djao OD, Daligault H, Lapidus A, Lucas S, Hammon N, Nolan M, Tice H, Cheng JF, Han C, Tapia R, Goodwin L, Pitluck S, Liolios K, Ivanova N, Mavromatis K, Mikhailova N, Pati A, Chen A, Palaniappan K, Land M, Hauser L, Chang YJ, Jeffries CD, Rohde M, Spring S, Sikorski J, Goker M, Woyke T, Bristow J, Eisen JA, Markowitz V, Hugenholtz P, Kyrpides NC, Klenk HP 2010 Complete genome sequence of *Methanoplanus petrolearius* type strain (SEBR 4847). Stand Genomic Sci 3:203–211. doi:10.4056/sigs.1183143. 21304750 10.4056/sigs.1183143 PMC3035365

[5] Tatusova T, Ciufo S, Fedorov B, O’Neill K, Tolstoy I 2014 RefSeq microbial genomes database: new representation and annotation strategy. Nucleic Acids Res 42:D553–D559. doi:10.1093/nar/gkt1274. 24316578 PMC3965038

[6] Anderson IJ, Sieprawska-Lupa M, Lapidus A, Nolan M, Copeland A, Glavina Del Rio T, Tice H, Dalin E, Barry K, Saunders E, Han C, Brettin T, Detter JC, Bruce D, Mikhailova N, Pitluck S, Hauser L, Land M, Lucas S, Richardson P, Whitman WB, Kyrpides NC 2009 Complete genome sequence of *Methanoculleus marisnigri* Romesser et al. 1981 type strain JR1. Stand Genomic Sci 1:189–196. doi:10.4056/sigs.32535. 21304656 PMC3035220

[7] Shimizu S, Ueno A, Tamamura S, Naganuma T, Kaneko K 2013 *Methanoculleus horonobensis* sp. nov., a methanogenic archaeon isolated from a deep diatomaceous shale formation. Int J Syst Evol Microbiol 63:4320–4323. doi:10.1099/ijs.0.053520-0. 23832970

[8] Rivard CJ, Smith PH 1982 Isolation and characterization of a thermophilic marine methanogenic bacterium, *Methanogenium thermophilicum* sp. nov. Int J Syst Bacteriol 32:430–436. doi:10.1099/00207713-32-4-430.

[9] Imachi H, Sakai S, Nagai H, Yamaguchi T, Takai K 2009 *Methanofollis ethanolicus* sp. nov., an ethanol-utilizing methanogen isolated from a lotus field. Int J Syst Evol Microbiol 59:800–805. doi:10.1099/ijs.0.003731-0. 19329610

[10] Bankevich A, Nurk S, Antipov D, Gurevich AA, Dvorkin M, Kulikov AS, Lesin VM, Nikolenko SI, Pham S, Prjibelski AD, Pyshkin AV, Sirotkin AV, Vyahhi N, Tesler G, Alekseyev MA, Pevzner PA 2012 SPAdes: a new genome assembly algorithm and its applications to single-cell sequencing. J Comput Biol 19:455–477. doi:10.1089/cmb.2012.0021. 22506599 PMC3342519

[11] Seemann T 2014 Prokka: rapid prokaryotic genome annotation. Bioinformatics 30:2068–2069. doi:10.1093/bioinformatics/btu153. 24642063

